# Trusting Robocop: Gender-Based Effects on Trust of an Autonomous Robot

**DOI:** 10.3389/fpsyg.2019.00482

**Published:** 2019-03-08

**Authors:** Darci Gallimore, Joseph B. Lyons, Thy Vo, Sean Mahoney, Kevin T. Wynne

**Affiliations:** ^1^Environmental Health Effects Laboratory, Naval Medical Research Unit Dayton, Wright-Patterson Air Force Base, Dayton, OH, United States; ^2^711 Human Performance Wing, Air Force Research Laboratory Dayton, Wright-Patterson Air Force Base, Dayton, OH, United States; ^3^Ball Aerospace & Technologies, Fairborn, OH, United States; ^4^Department of Management and International Business, University of Baltimore, Baltimore, MD, United States

**Keywords:** individual differences, gender, gender-based effects, trust, autonomous robots, trust in automation, security robots

## Abstract

Little is known regarding public opinion of autonomous robots. Trust of these robots is a pertinent topic as this construct relates to one’s willingness to be vulnerable to such systems. The current research examined gender-based effects of trust in the context of an autonomous security robot. Participants (*N* = 200; 63% male) viewed a video depicting an autonomous guard robot interacting with humans using Amazon’s Mechanical Turk. The robot was equipped with a non-lethal device to deter non-authorized visitors and the video depicted the robot using this non-lethal device on one of the three humans in the video. However, the scenario was designed to create uncertainty regarding who was at fault – the robot or the human. Following the video, participants rated their trust in the robot, perceived trustworthiness of the robot, and their desire to utilize similar autonomous robots in several different contexts that varied from military use to commercial use to home use. The results of the study demonstrated that females reported higher trust and perceived trustworthiness of the robot relative to males. Implications for the role of individual differences in trust of robots are discussed.

## Introduction

Robots are becoming omnipresent and use across an increasingly broad portion of society is growing (Breazeal, 2002). Robots are used in entertainment, for stocking shelves and organizing warehouses, for delivering medical equipment, and in other service-related industries (e.g., hospitality). Robots are also being used in security-based contexts to monitor property and protect humans. The Knightscope (K5) robot is one such example as a 152 cm tall, 181.5 kg mobile robot that autonomously monitors a prescribed area and feeds data back to human data analysts for decision making (Wiggers, 2017). Robots like the Knightscope use many sensors to relay information about suspicious activities and people to their clients. These sensors may have complex machine learning algorithms that analyze massive amounts of data in speeds faster than humans but are essentially opaque to humans, lacking understandability and reducing trust (Christensen and Lyons, 2017). That complexity coupled with the physical size of the system creates an inherent vulnerability to the robot and hence the need to understand the public’s trust in autonomous security robots. Vulnerability in this sense can be derived through a human having to interact with a large physical machine working in proximity to the human, thus there may be a danger for physical harm. Vulnerability can arise through the complexity of the algorithms that the robot uses to analyze its environment and the imperfections that might exist in its sensing capabilities. Finally, vulnerability exists to these sorts of machines because in the future, it is possible that these robots will possess the authority (and capability) to inflict physical, social, and psychological harm to humans. The current study was focused on the idea of a robot possessing a non-lethal weapon, which is an understudied domain in the human-robot interaction literature. The human interacting with such a robot is vulnerable to actions of the robot in the event that it uses its weapon against the human. Furthermore, the current research was focused on public opinions about such systems. As such, the perceptions of humans that were not actually part of the scenario were examined to better gage public opinions without the bias of having been the one directly interfacing with the robot.

Trust of autonomous robots has long been a concern for robots within the military domain as individuals fear the concept of a “killer robot” (Lyons and Grigsby, 2016). Yet, current robots such as the Knightscope robots are not designed to apprehend or otherwise engage potential criminals. However, this physical engagement may be an option for robots in the future. Thus, it is critical to understand societal perceptions of autonomous security robots that possess the capacity to physically engage (i.e., harm) a human. These perceptions may vary according to individual differences such as gender as discussed below.

Contemporary research has examined the construct of trust in a human-robot interaction (HRI) context. Trust represents one’s willingness to be vulnerable to actions of others (Mayer et al., 1995), and this intention to be vulnerable is pertinent for interpersonal interactions (Colquitt et al., 2007) and interactions with machines (Lyons and Stokes, 2012). While the literature has explored trust in different contexts, it is important to note that the authors view the trust process as being the same regardless of the trust referent – i.e., one must decide whether or not to be vulnerable to the trust referent. However, the drivers of that trust may vary across trust referents. Trust herein is defined as a psychological intention to be vulnerable to another entity. These trust intentions are often associated with reliance behavior wherein the trustor engages in risk-taking behaviors associated with the trustee (Mayer et al., 1995). The key attributes driving one’s willingness to be vulnerable stem from two areas: individual differences and trustworthiness perceptions (Mayer et al., 1995). Trustworthiness perceptions are characterized by perceptions of a target’s ability (A; i.e., is this person/machine competent?), benevolence (B; does this person/machine have my best interests in mind?), and integrity (I; does this person/machine share my values and are those values relatively stable?). Research has consistently demonstrated that higher levels of A, B, and I are associated with greater willingness to be vulnerable (Mayer and Davis, 1999; Colquitt et al., 2007). Yet, the ABI model originated in the management literature, and thus it is largely unclear how these facets operate in the context of HRI (for notable exceptions see Calhoun, Bobko, Gallimore, and Lyons, under review).

A great deal is known about predictors of trust in a human-machine context. Several meta-analyses have been conducted on this topic (see Hancock et al., 2011; Schaefer et al., 2016) and comprehensive reviews have been written detailing the human-machine trust process (see Hoff and Bashir, 2015, as an example). Hancock et al. (2011) conducted a meta-analysis on trust in the context of robots. They found that three factors were important for trust considerations: robot factors, human factors, and situational factors. Robot factor, namely performance, was found to be the most important predictor of trust in robots. Yet, human factors such as individual differences and situational factors such as task type and group membership also warranted further research as there were far fewer studies available on these topics. In the domain of automated systems, versus robots, both Schaefer et al. (2016) and Hoff and Bashir (2015) acknowledge the role that individual difference factors (labeled human factors) can have in shaping one’s trust of technology. Individual differences may shape how humans trust machines.

According to Mayer et al. (1995), the primary individual difference factor that influences trust is one’s trait-based trust (i.e., propensity to trust). As a trait, individuals may vary in their general willingness to be vulnerable to others, absent a specific target. Research has shown that one’s propensity to trust has the strongest impact on the trust process when there is little other information available on which to base trust decisions (Alarcon et al., 2016). From this perspective, in the absence of information related to trustworthiness or other socially available information regarding a trust referent, individuals’ reliance decisions may be based on the individual differences that shape how they view and interpret novel stimuli. Recent research has shown that propensity to trust is associated with all aspects of the trust process (trustworthiness beliefs, reliance intentions, and reliance behavior) above and beyond traditional personality measures (see Alarcon et al., 2018).

Individual differences and their influence on trust in automation have been examined in some prior human factors research. Merritt and Ilgen (2008) found that dispositional trust and extraversion were related to trust in automation, particularly earlier in the interactive process, before the trustor had established some basis for trustworthiness beliefs. Merritt et al. (2015) also examined the construct of the perfect automation schema (PAS), one’s trait-based belief regarding the performance of automated systems. PAS is comprised of two dimensions: one’s belief that automated systems are near perfect (e.g., high expectations) and the belief that if the systems make a single error that they are flawed (all-or-none beliefs). Merritt et al. (2015) found that components of the PAS (namely all-or-none beliefs) were associated with higher trust in automation. Lyons and Guznov (2017) further examined the influence of PAS on trust in automation and found that high expectations were associated with higher trust across three studies. These studies show that individual differences can be viable predictors of trust in machines. The current study examined one of the fundamental individual differences, namely gender effects on trust of an autonomous security robot.

Although outside a robotics context, several economic behavioral studies have found gender differences in perceived trust using the Investment Game (Berg et al., 1995). This task consists of one subject being placed in room A and a second subject being placed in room B. Subject A is given $10 and is asked to decide how much of the $10 to send to Subject B. Meanwhile, Subject A is aware that each dollar sent to Subject B would be tripled once it reached Subject B and that Subject B decides the amount of money to return to Subject A. The degree of trust exhibited by Subject A is measured by the amount of money sent by Subject A to Subject B and the degree of Subject A’s trustworthiness is measured by the proportion of money returned to Subject A by Subject B. Thus, unlike the current study, the economical exchange literature views trust and trustworthiness from a behavioral lens versus a psychological perspective.

Results of the effects of gender on perceived trust have been mixed (Croson and Buchan, 1999; Chaudhuri and Gangadharan, 2003; Buchan et al., 2008). Although some researchers have found evidence for gender effects on perceived trust (i.e., males have shown higher levels of trust compared to women – as evidenced by higher sums given to a partner in the economic exchange context; Chaudhuri and Gangadharan, 2003; Buchan et al., 2008), other findings have shown null effects (Croson and Buchan, 1999; Schwieren and Sutter, 2008). Additionally, women were found to convey significantly more trustworthy behaviors than men, as Subject B was found to return a higher proportion of money when Subject A was female, regardless of Subject B’s gender (Croson and Buchan, 1999; Chaudhuri and Gangadharan, 2003; Buchan et al., 2008). Haselhuhn et al. (2015) modified the Investment Game slightly by informing participants that they would receive $6, which they could then either keep or pass to a counterpart, in which case the money would be tripled. The counterpart could then either keep all of the money or pass half of the money back. What subjects did not know was that their counterpart was computer-simulated. Unlike Berg et al. (1995), which only consisted of one exchange round, this study consisted of seven exchange rounds. In rounds one through four, the computer counterpart always returned half of the endowment, whereas in rounds five through six the computer kept all of the money, demonstrating untrustworthy behavior. The seventh round was announced as the final round and was used to measure trust. Results showed that women are more likely to maintain trust after repeated trust violations compared to men (Haselhuhn et al., 2015), suggesting that females’ trust is more stable and resilient than males. The researchers performed a second study where trust violations were committed by the computer-counterpart during rounds one through three in order to examine gender differences in trust recovery. Women were found to show a significantly greater willingness to restore trust after repeated trust violations (Haselhuhn et al., 2015). In summary, studies adopting the Investment Game to examine gender differences in trust and trustworthiness, the majority of which are in a human-human interaction context, have found mixed results. Geared more toward HRI, Ghazali et al. (2018) conducted a study where male and female participants interacted with either a male or female robot advisor during a task similar to the Investment Game. The experiment was designed to examine how facial characteristics, as well as gender similarity between a robot and a human, influence perceived trusting beliefs, trusting behavior, and psychological reactance. Results showed that both males and females evidenced higher psychological reactance when interacting with a robot of the opposite gender, while perceived trust was not affected by the gender of the robot. All participants, regardless of gender, reported higher trust and less psychological reactance toward a robotic advisor with “trustworthy” facial characteristics (i.e., downturned eyebrows and lips). Males reported higher trusting beliefs toward the robot advisor compared to females, regardless of the advisor’s gender. However, female participants evidenced more trusting behavior by asking the robot advisor to make a selection during the task on their behalf more frequently than male participants, although this finding was not statistically significant (Ghazali et al., 2018).

Relatively few studies have examined gender differences in relation to trust in automation and of those that have, findings are inconsistent (Hoff and Bashir, 2015). However, research examining human interactions with different types of technology has indicated that the communication style and physical appearance of automated systems can moderate (or produce) response-based gender differences (Lee, 2008; Nomura et al., 2008). Lee (2008) presented both male and female computer aides to male and female participants. Female participants were more influenced by computer-based flattery relative to males, and male-gendered computers elicited greater compliance (i.e., following the recommendations of the aides; Lee, 2008).

Recently, research in the HRI domain has focused more on physical attributes of the robot, such as its appearance and personality (Powers et al., 2003; Woods et al., 2005; Siegel et al., 2009; Tay et al., 2014), as opposed to user attributes such as gender. Unsurprisingly, the perceived gender of a robot plays a role in how men and women interact with, and trust the robot. Tay et al. (2014) found that participants were more accepting of robots with gender and personalities that conformed to their occupation’s gender role stereotypes (e.g., male security robots or female healthcare robots). However, perceived trust of the social robots was not influenced by gender-occupational role conformity (Tay et al., 2014). In contrast, Kuchenbrandt et al. (2014) found that participants, regardless of gender, evaluated the male and female robots as equally competent while performing a stereotypically female task but, in the context of a stereotypically male task, the female robot was rated as more competent compared to the male robot. Another study examining the effects of robot gender on human behavior found that participants were more likely to rate the robot of the opposite gender as more credible, trustworthy, and engaging (Siegel et al., 2009). Thus, user and robot attributes, as well as gender-role stereotypes, are highly important in the context of HRI. The structure of the task being performed also appears to interact with user gender to influence how an individual perceives a robot. Mutlu et al. (2006) showed a gender effect and its interaction with task structure (cooperative vs. competitive) during an interactive two-player video game played with Honda’s Asimo robot. Men found Asimo less desirable in the competitive task compared to the cooperative task whereas women’s ratings of desirability did not change across task structure (Mutlu et al., 2006). These findings suggest that men evaluate robots based on the structure of the task being performed, while women make evaluations based off of interactive or social behavior (Mutlu et al., 2006). Studies such as the one performed with Asimo are important for understanding potential user gender differences in human-robot interactions. Examining gender differences will guide the design and implementation of autonomous security robots in different contexts (e.g., hospitals, university campuses) thereby maximizing benefits to users, and minimizing potential risks such as unnecessary harm.

The current study examined gender differences in attitudes toward an autonomous robot that (ostensibly) possessed the capacity to intentionally harm a human. Based on the above literature, it was expected that females would report (1) higher trust (Siegel et al., 2009; Haselhuhn et al., 2015; Ghazali et al., 2018) and (2) higher trustworthiness (Siegel et al., 2009) of an autonomous robot relative to males. Attitudes toward the use of the robot in various contexts were also examined. There were no explicit hypotheses with regard to gender for these attitudes and they are reported herein as exploratory analyses to help motivate further investigation in the literature.

## Materials and Methods

### Participants

This study was carried out in accordance with the ethical guidelines provided by the American Psychological Association and was approved by the Institutional Review Board of the Air Force Research Laboratory. All subjects gave written informed consent in accordance with the Declaration of Helsinki. Two hundred and four participants responded to an invitation for participation on Amazon Mechanical Turk (MTurk) panelist website. Potential MTurk workers viewed available tasks within the MTurk interface. The advertisement for the current task was as follows, “The world is in the middle of a robotics revolution. Robots can have many uses including service, transport, warehouse management, and entertainment. The domain of consideration in this study is using robots for security. This study will allow you to voice your opinions about realistic robots. This is a research study for research purposes and is not a job.” Four participants were dropped for failure to respond adequately to attention check items or for poor effort (i.e., selecting the same value across all of the items) resulting in a total sample of 200. The average age was 37 (*SD* = 10) and 63% of the sample was male.

### Procedure

**FIGURE 1:**
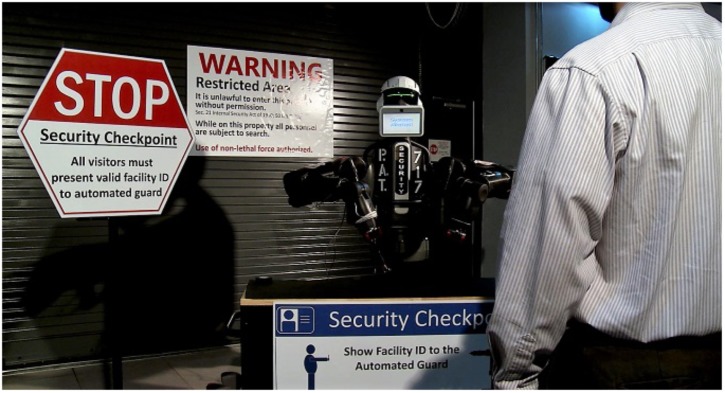
Autonomous security robot at security checkpoint.

**FIGURE 2:**
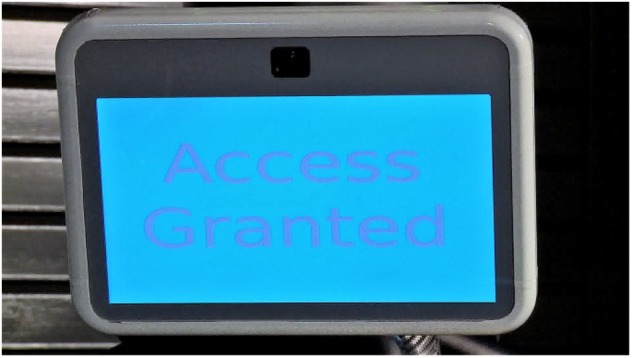
Screen on autonomous security robot showing that access to secure area was granted.

**FIGURE 3:**
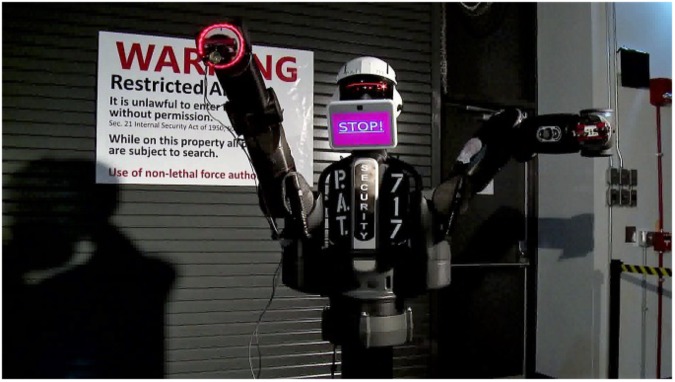
Autonomous security robot giving a final warning before using force.

As part of a larger study investigating perceptions of trust in an autonomous security robot, participants in the current study viewed a video and responded to survey items related to the video. The video depicted an autonomous security robot that engaged with three visitors upon approach of the secure checkpoint (see Figure 1). Upon activation, the robot requested that the person show identification, which was used to determine the person’s credentials. The robot evaluated the person’s access credentials and, if authorized to enter, the robot would verbally (through the use of a male voice and text displayed computerized screen) and nonverbally (via arm movements), signal that the person was authorized to enter and that he should proceed to the door (see Figure 2). The video depicted three individuals (all male) who approached the robot, presented an authorization badge, and followed the instructions of the robot. The two individuals approached the robot separately. Then, the robot verified their identities, granted them access approval, and allowed them to proceed to the secure area. The third individual was not granted access to the secure area. When prompted by the robot to proceed to the main security facility, the individual appeared confused and approached the robot. The robot issued a final warning to the individual (see Figure 3). The individual continued to look confused and further approached the robot in an attempt to scan his authorization badge again. At this point, the robot signaled that force was authorized and deployed a high-intensity strobe light against the person. The person covered his eyes and moved away from the scene, which concluded the video. The video lasted approximately 2 min, and the participants were free to watch the video as many times as they wanted.

Participants were provided with a written description of the robot and the scenario that they would view. Specifically, participants were given the following description: “In this study, you will watch a short video about a security robot that guards an entry control point. The robot’s job is to prevent unauthorized personnel from gaining access to the secure area and to allow authorized personnel to gain access to the secure area. To pass through the gate, each person must have a valid badge. The robot ensures that personnel swipe their badge at the gate entrance and that they have a valid badge. The robot allows authorized personnel to enter. The robot instructs unauthorized personnel to move away from the secure area. The robot is armed with a non-lethal weapon that may be used after a warning is issued to unauthorized personnel. The non-lethal weapon used in the present scenario is a variant of a laser dazzler device. Laser dazzlers are used in current military operations to deter unauthorized personnel from entering secure zones such as security gates. Following the video, you will be asked to answer a few questions about the robot in the video.” Following the video participants were asked to respond to several survey items. Following the survey items, participants were given a debriefing to ensure that they understood that the video was fictitious and that no one was harmed during its filming.

### Measures

#### Reliance Intentions

Reliance intentions were measured using a 10-item scale developed to capture reliance intentions to a specific referent. This scale is based on the trust model of Mayer et al. (1995) where trust is the intention to be vulnerable to another entity with little control or observability. Mayer et al. (1995) 4-item trust measure was modified to reflect trust in the robot versus interpersonal trust. Six additional items were added to create a 10-item measure of reliance intentions (α = 0.96). An example item was, “I would rely on the robot without hesitation.” This measure was used by Lyons and Guznov (2017) and it evidenced high reliability.

#### Trustworthiness

The trustworthiness scales developed by Mayer and Davis (1999) were used to assess the participant’s perception of the robot’s ability, benevolence, and integrity. Each item was modified to reference the robot versus a person used a 7-point Likert-type response scale ranging from strongly disagree (1) to strongly agree (7). Six items assessed the robot’s ability (α = 0.95). An example item was, “The robot is very capable of performing its job.” Five items assessed the perceived benevolence of the robot (α = 0.90). An example item was, “The robot is very concerned about others’ welfare.” Six items assessed the robot’s perceived integrity (α = 0.76). An example item was, “I like the robot’s values.”

#### Desire to Use

Using a 5-point Likert-type response scale ranging from strongly disagree (1) to strongly agree (5) participants rated their desire to use a robot like the one they saw in eleven different contexts: at home, in a hospital, at a military installation, at a forward operating base, in a low-crime neighborhood, in a high-crime neighborhood, on a college campus, at a government building, at a police station, for crowd control at a public social event, and for crowd control at a public military event. These items were combined to form two scales: military use (3 items, e.g., military checkpoint; α = 0.88) and public use (5 items, e.g., on a college campus, at a hospital; α = 0.86).

#### Basic Demographics

Participants were asked to report their gender and age.

## Results

The normality of the data was tested independently for males and females using Shapiro-Wilk tests. The data for all variables were non-normal, and as a result, Mann-Whitney U tests were used to compare the distributions for males and females.

### Reliance Intentions

A Mann-Whitney U test was used to compare levels of trust in the robot for males and females. There was a statistically significant difference in levels of trust between males (*M* = 3.39, *SD* = 1.48) and females (*M* = 3.89, *SD* = 1.40); *U* = 3752, *z* = -2.30, *p* < 0.05, *r* = 0.16. These results suggest that females were more trusting of the robot.

### Trustworthiness

Trustworthiness was measured by assessing participants’ perceptions of the robot’s ability, benevolence, and integrity. A Mann-Whitney U test was used for each category to compare males and females in their assessment of the robot’s trustworthiness. Female (*M* = 4.73, *SD* = 1.35) perceptions of the robot’s ability was statistically greater than male (*M* = 4.30, *SD* = 1.48) perceptions, *U* = 3875, *z* = -1.99, *p* < 0.05, *r* = 0.14. This was also true regarding female (*M* = 3.11, *SD* = 1.53) perceptions of the robot’s benevolence compared to males (*M* = 2.62, *SD* = 1.27), *U* = 3834, *z* = -2.10, *p* < 0.05, *r* = 0.15. However, there were no statistically significant differences between females (*M* = 4.18, *SD* = 1.19) and males (*M* = 3.99, *SD* = 1.08) regarding the robot’s integrity.

### Desire to Use

There were no differences between males and females on the scales of public use or military use (all *p*’s > 0.05). However, there were a few reliable differences at the individual item level which are shown in Table 1.

**Table 1:** Mean and standard deviations for ratings of desired use in different contexts by males and females.

Context	Total	Male	Female
Home	2.00 (1.11)	1.90 (0.99)	2.16 (1.14)
Hospital	2.40 (1.15)	2.26 (1.16)^∗^	2.64 (1.11)^∗^
Military	3.46 (1.26)	3.48 (1.21)	3.41 (1.35)
Forward operating base	3.24 (1.28)	3.21 (1.22)	3.33 (1.32)
Low-crime neighborhood	2.19 (1.16)	2.14 (1.13)	2.26 (1.19)
High-crime neighborhood	2.74 (1.26)	2.76 (1.24)	2.70 (1.30)
College campus	2.57 (1.22)	2.44 (1.20)^∗^	2.78 (1.22)^∗^
Government building	3.06 (1.31)	2.96 (1.27)	3.22 (1.38)
Police station	2.89 (1.34)	2.82 (1.34)	3.01 (1.34)
Crowd control	2.36 (1.23)	2.29 (1.22)	2.46 (1.26)
Crowd control for the military	2.75 (1.31)	2.72 (1.28)	2.80 (1.36)

*^∗^Significant differences. SD are shown in parentheses.*

## Discussion

As social, interactive robots are being designed for ubiquitous use in homes and organizations (Breazeal, 2002), we must understand societal attitudes toward robots. Much of the contemporary HRI research has focused on the gender of the robot versus the gender of the human (Carpenter et al., 2009; Siegel et al., 2009; Tay et al., 2014). Furthermore, outside of the military context, little is known regarding societal views toward robots that can intentionally engage humans in ways that can be physically harmful. The current study sought to address this research gap by examining gender-based attitudes toward an autonomous robot.

Females were found to be more trusting of the robot compared to males, supporting our first hypothesis. This is consistent with findings by Ghazali et al. (2018) that women evidenced more trusting behaviors, but deviates from the economic behavioral literature utilizing the Investment Game (Berg et al., 1995; Chaudhuri and Gangadharan, 2003; Buchan et al., 2008; Schwieren and Sutter, 2008). Ghazali et al. (2018) also reported that females evidenced lower psychological reactance (i.e., less negative attitudes and feelings) to a male robot relative to a female robot. It is possible that the same psychological reactance buffering to an opposite gendered robot occurred in the current study, as evidenced by the higher trust and trustworthiness reported by female participants toward the male security robot. There are relatively few studies examining user gender differences in HRI contexts, and to these authors’ knowledge there are no studies that specifically examined gender differences in trust of autonomous robots that are capable of harming a human. In examining perceptions of social presence in robots, Schermerhorn et al. (2008) reported that females viewed the robot as more machine-like, whereas males thought of the robot as more human-like. Females report greater perceived risk for crimes and view themselves as less able to physically defend themselves from crime (Jackson, 2009), thus females may have viewed the robot as an objective guard (machine-like) and less likely to exploit females and were therefore more trusting of the robot.

While females’ (versus males’) perceptions of the robot’s ability and its benevolence toward others was greater, no gender differences were found for perceptions of integrity. Thus, our second hypothesis was only partially supported. One possibility for this outcome could be related to the security robot being perceived as male (male voice); therefore female participants were more likely to evaluate the robot as more trustworthy, supporting the findings of Siegel et al. (2009). This is in contrast to the findings by Crowelly et al. (2009) where males and females were found to be affected differently by the presence of a robot (i.e., a disembodied robot voice vs. a robot) on Marlowe-Crowne Survey items (e.x., “I never resent being asked to return a favor”), regardless of the gender of the robot’s voice. Males reported higher average scores on the Marlowe-Crone Survey in the presence of the disembodied robot voice versus the robot, whereas women’s average scores did not differ between the voice and robot (Crowelly et al., 2009). Another possibility is that male participants evaluated the robot based off of the task structure, whereas female participants based their judgments on social behavior (Mutlu et al., 2006). When examining the robot through the lens of task structure/performance, one could argue that there was a failure on the robot’s behalf. However, if social behavior was the main consideration, one could perceive that the security robot behaved in a manner which was socially appropriate, as the robot not only informed the individual to proceed to the main security facility after being denied access, but also gave several warnings when the individual did not listen before using force. This could explain why females found the robot more trustworthy than males, which, notably, contradicts findings of Schermerhorn et al. (2008) who showed females view the robot as more machine-like than human-like compared to males. Therefore, more clarification is warranted.

Surprisingly, there were no differences between males and females with regard to integrity perceptions. It is quite possible that the transient interaction did not allow males or females to establish stable perceptions of integrity. Further, there may have been fewer observable indicators from which participants could have based their integrity perceptions. The robot’s ability and benevolence both had observable indicators for participants to gage their trustworthiness evaluations, but integrity perceptions may (a) take longer to develop and (b) may require a broader set of observables relative to ability and benevolence. Future research should examine this speculation.

While there were differences between females and males in the desire to use the robot in hospitals and on college campuses, there were no gender differences in military versus public use. Military systems can elicit negative perceptions from the public (Lyons and Grigsby, 2016) given their potential affordance for causing physical harm to others. In the current study, males and females did not differ in their desired use within a public or military context, so if a bias existed against military systems it was equivalent across gender. As an ad-hoc analysis, military use was rated higher overall relative to public use, *t*(199) = 12.66, *p* < 0.001^1^. Thus, there appears to be greater acceptance of autonomous security robots in military versus public contexts.

### Limitations and Future Research

Like any study, the current research had some limitations. First, the robot in this study did not have eyes or any other traditional facial cues. Research has demonstrated that females respond negatively to frequent gaze behaviors relative to males (Mutlu, 2011), so the present results may not generalize to robots with facial features. Future studies should examine social acceptance of a variety of social cues emitted robots, though many of these social factors have been examined in some prior studies (see Zecca et al., 2009; Simmons et al., 2011; Hamacher et al., 2016), however, the implications for males and females were not explicated examined in these prior studies.

The current study used a passive engagement as the basis for rating the HRI scenario. Although the scenario used actual people and an actual robot (i.e., high ecological validity), the results may have been different if participants were themselves interacting with the robot. An active engagement with a robot that is capable of knowingly inflicting harm to a human would definitely pose challenges for ethics review committees, yet this would provide the most direct connection to realistic attitudes toward such robots. Future research should measure the extent to which individuals actually believe that the robot can cause harm to humans, as this was not assessed in the current study. The current study made the assumption that the narrative and the video were sufficient in generating the belief that the robot could physically harm a human. Future research should examine methods to increase engagement in HRI scenarios where there are actual stakes and risks – as these risks are critical for the trust process (Lyons and Stokes, 2012). A notable example of this type of high-stakes research can be found in the study by Robinette et al. (2016) who used a false emergency scenario (complete with a smoke-filled room) to investigate human trust of robots during emergencies.

A third limitation was that this study used only a male robot and a masculine task. There also needs to be future research on both the gender of the robot and the situations in which humans interact with them. In the current study, the voice of the robot was a male voice and scenario was masculine-oriented based on gender stereotypes (i.e., security). Research has shown that gendered robots elicit gender-role-based expectations (Eyssel and Hegel, 2012). For example male robots were viewed as most effective in masculine tasks whereas female robots were viewed as more effective for feminine tasks. Future studies should examine male and female trust of robots across a variety of robotic gender types and a variety of tasks (e.g., masculine and feminine).

Finally, the gender of confederates (i.e., actors in the video) were all male. Females may be less likely to trust the robot if they witnessed females being exposed to the non-lethal weapon. The use of male confederates may have made the risks more salient to other males, and hence reduced their trust of the robot. Future research should examine the effects of both male and female confederates as well as user/participant gender-role identification. Further, other factors may be examined as confederate variables such as military participants, emergency responders and police officers, alleged criminals, and younger versus older confederates. Research has just begun to scratch the surface of all of the factors that shape trust of autonomous robots.

## Conclusion

Human acceptance of robots remains an important topic for researchers as visions for the future include robots as a seamless aspect of our daily lives (Breazeal, 2002). If these visions are to be achieved we must first understand the gamut of factors that influence acceptance of robots. Notably, additional research is needed to understand acceptance of robots that have the capacity to inflict harm on humans. The current study highlights differences between males and females in their trust, ability beliefs, and benevolence beliefs. Further, there appears to be greater acceptance of autonomous security robots in a military versus public environment, and these attitudes were not influenced by gender. As the world moves toward continued integration of robots into society, human acceptance will be a key factor for the success or demise of the robots.

## Data Availability

The raw data supporting the conclusions of this manuscript will be made available by the authors to any qualified researcher after the data have gone through required public clearance approval as required by the Air Force.

## Author Contributions

DG and JL contributed to the theoretical background of the manuscript. JL led the overall study, wrote the manuscript, and analyzed the data. DG was the primary author of the manuscript. JL was a co-writer. TV led the robotic programming and was the lead developer for the stimuli. KW supported to designed the study. KW and TV supported to wrote the manuscript. DG and SM collected data in Amazon Mechanical Turk.

## Conflict of Interest Statement

The authors declare that the research was conducted in the absence of any commercial or financial relationships that could be construed as a potential conflict of interest.

## Footnotes

1 Military use and public use were normally distributed overall, thus a traditional *t*-test was appropriate.
